# Supplementary material for: DMRforPairs: identifying Differentially Methylated Regions between unique samples using array based methylation profiles
Source: BMC Bioinformatics. 2014 May 15;15:141. doi: 10.1186/1471-2105-15-141 (PMC4046028; doi:10.1186/1471-2105-15-141)
Supplement: Additional file 2 — DMRforPairs output for the comparison of A431-MCF7 and NA17018-NA17105. Please start from the HTML files in each folder. Available via the BMC Bioinformatics website. [file 1471-2105-15-141-S2.zip › 1394847754114233_MOESM2_ESM/A431_MCF7/figures/12542.pdf]

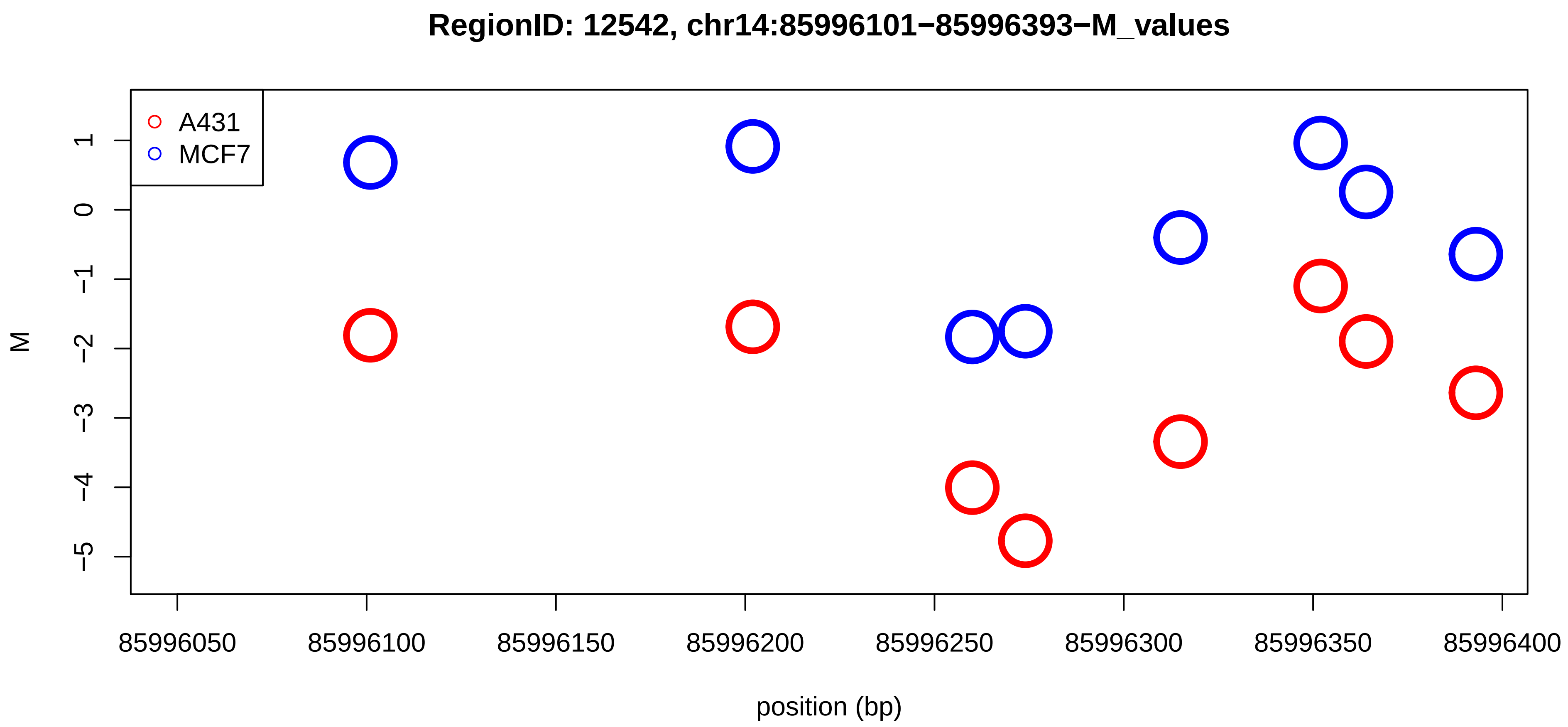
RegionID: 12542, chr14:85996101−85996393−M_values
A431
MCF7
M
1
0
−1
−2
−3
−4
−5
85996050
85996100
85996150
85996200
85996250
85996300
85996350
85996400
position (bp)

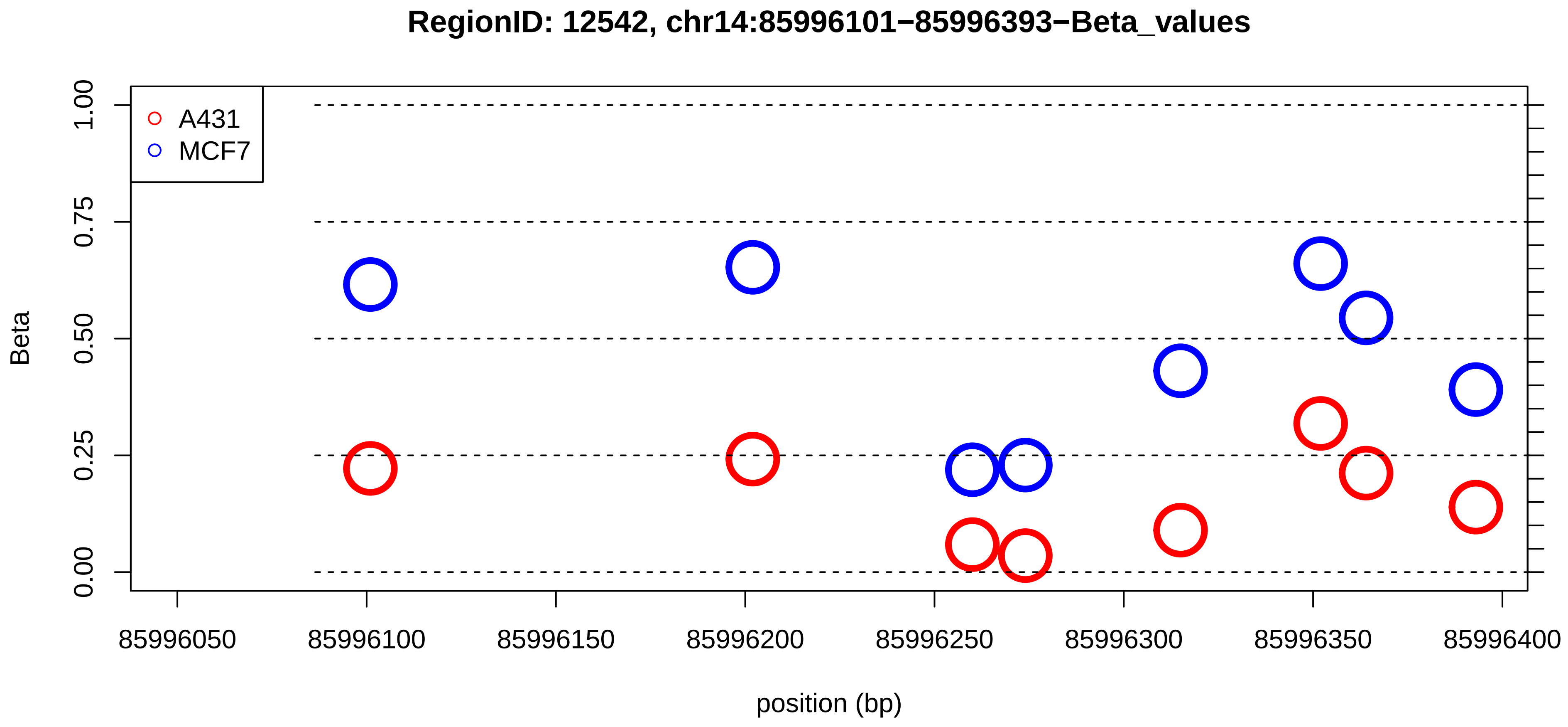
RegionID: 12542, chr14:85996101−85996393−Beta_values
A431
MCF7
Beta
1.00
0.75
0.50
0.25
0.00
85996050
85996100
85996150
85996200
85996250
85996300
85996350
85996400
position (bp)

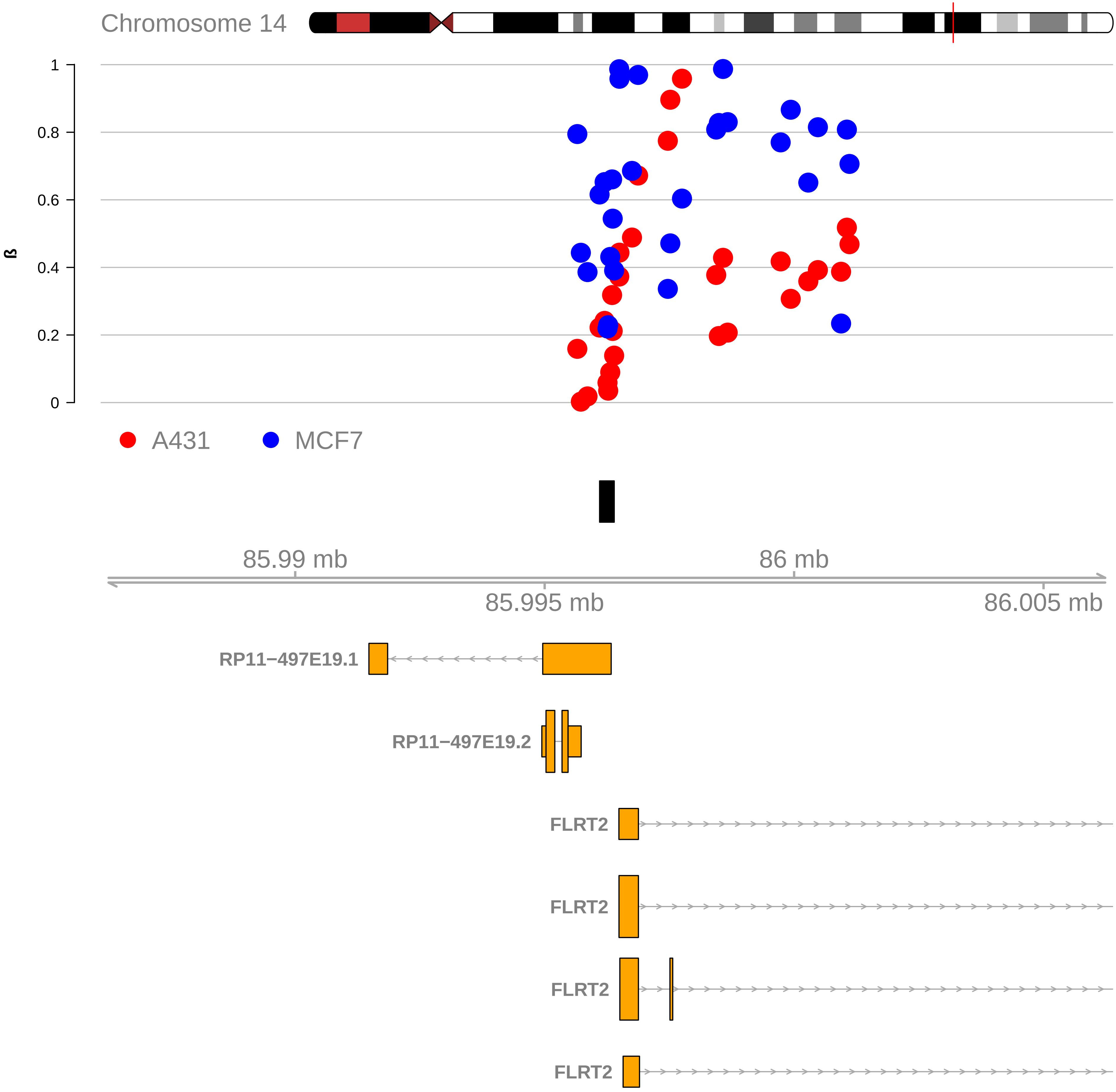

Chromosome 14
1
0.8
0.6
0.4
0.2
0
ß
A431
MCF7
85.99 mb
86 mb
85.995 mb
86.005 mb
RP11−497E19.1
RP11−497E19.2
FLRT2
FLRT2
FLRT2
FLRT2
